# An investigation into an outbreak of pancytopenia in cats in the United Kingdom

**DOI:** 10.1111/jvim.16615

**Published:** 2023-01-07

**Authors:** Barbara Glanemann, Karen Humm, Camilla Pegram, Daniel L. Chan

**Affiliations:** ^1^ Department of Clinical Science and Services Royal Veterinary College Hatfield UK; ^2^ Department of Pathobiology and Population Sciences Royal Veterinary College Hatfield UK

**Keywords:** diacetoxyscirpenol, neutropenia, T‐2/HT‐2 mycotoxin, thrombocytopenia

## Abstract

**Background:**

In spring 2021 increasing numbers of cats presenting with severe pancytopenia were noted in United Kingdom (UK).

**Objective:**

To describe process and outcome of the investigation performed into the outbreak of pancytopenia in cats.

**Animals:**

Five hundred and eighty client owned cats that presented with severe bi‐ or pancytopenia of unknown cause.

**Methods:**

Real‐time data collection was performed by an online registration forum available to all veterinary surgeons in UK. Data collected included demographics, clinicopathological findings, diagnostic testing, dietary and drug history, outcome and COVID household status. Mycotoxicological feed analysis was performed on feed samples of 3 diets frequently mentioned in the database and 3 control diets.

**Results:**

Five hundred and eighty cats presented to 378 veterinary practices were included for analysis. Case fatality rate was 63.3%. Dietary history was available for 544 (93.8%) cats, of which 500 (86%) were fed 1 of 3 diets (which were recalled midinvestigation). 54 (9.3%) cats were not fed a recalled product, with diet information unknown in 26 (4.5%) cats. Analysis of feed samples revealed concentrations of hematotoxic trichothecene T‐2/HT‐2 mycotoxins greater than recommended by the European Commission in 5/7 recalled diet samples but in none of control diet samples. The trichothecene mycotoxin diacetoxyscirpenol (DAS) was detectable in all recalled diet samples but not in any of control samples.

**Conclusion and Clinical Importance:**

Contaminated‐feed induced trichothecene mycotoxicosis should be considered as a differential diagnosis for pancytopenia in cats.

**Abbreviations**
DAS: diacetoxyscirpenol
FELV: feline leukemia virus
FIV: feline immunodeficiency virus
FPLV: feline panleukopenia virus
FSA: Food Standard Agency
LOAEL: lowest observed adverse effect level
NOAEL: no observed adverse effect level; UK, United Kingdom

## 1 INTRODUCTION

Pancytopenia in cats, characterized by moderate to severe neutropenia, thrombocytopenia and anemia, is caused either by peripheral destruction or consumption of blood cells, or a combination of these, or by a failure of the bone marrow to produce.^1^ Causes of pancytopenia in cats include infectious diseases (eg, leukemia, immunodeficiency, and panleukopenia virus infection), neoplasia (eg, lymphoma, leukemia, hemophagocytic syndrome), toxicosis (eg, drugs, radiation), deficiencies in essential minerals and vitamins (eg, cobalamin deficiency), immune‐mediated, and idiopathic disease.^2^, ^3^, ^4^, ^5^, ^6^ During the spring of 2021 a suspected outbreak of pancytopenia of unknown cause in cats was recognized in the United Kingdom (UK). Severe pancytopenia in cats with no identifiable cause has been a rare presentation to our tertiary referral hospital (Queen Mother Hospital for Animals, Royal Veterinary College, UK), being diagnosed only in 5 (0.13%) of 3895 cats presenting to the hospital and undergoing complete blood cell analysis over a 5‐year period (January 2015 to December 2019). Within a period of 4 weeks in spring 2021 7 cats, including cats that shared households, presented to the authors' hospital, with a few days' history of lethargy, anorexia, and bleeding tendencies. All cats had severe bi‐ or pancytopenia; and an underlying cause could not be identified despite thorough investigations. All 7 cats died or were euthanized because of ongoing hemorrhage. Additionally, advice enquiries from first opinion as well as other referral hospitals in regard to similar presentations were received at an unusually high frequency.

In the UK, clear guidance for the management of communicable disease outbreaks and for foodborne illness exist for illnesses affecting people.^7^, ^8^ However, the management of outbreaks of veterinary diseases has not been described in small animal practice. This report aims to describe both the investigation of the outbreak and its findings, and the affected cohort of cats.

## 2 MATERIAL AND METHODS

### 2.1 Questionnaire

To gather case related information formally, an online questionnaire (Supplementary information S1) was designed and published publicly on 24 May 2021 which allowed any veterinary practitioner to submit information about cats presenting with severe pancytopenia. The questionnaire was publicized through emails to members of the Veterinary Poisons Information Service, direct contact of American or European board‐certified specialists in internal medicine within the UK (addresses obtained from central databases; for example, ECVIM listing; www.ecvim-ca.org/specialist-listings) and members of veterinary medicine specialist interest groups including the Small Animal Medicine Society, the British Small Animal Veterinary Association, the Association of Veterinary Hematology and Transfusion Medicine and Diplomates of the American College of Veterinary Internal Medicine (listserv of the American College of Veterinary Internal Medicine). Letters explaining the nature of the investigation and containing the link to the questionnaire were also sent to veterinary trade journals.^9^

Data collected included date of presentation, signalment, duration and type of clinical signs, indoor/outdoor status, clinical pathological findings, bone marrow sampling details, feline immunodeficiency (FIV), feline leukemia (FeLV) and feline panleukopenia (FPLV) virus testing results, parasite control medication, details of any other recent medication or vaccination, cat litter type used, and dietary information for each cat affected. Details on owner household location (through the first part of the owner postcode), any history of COVID 19 infection in the household and whether any other household pets were affected were also gathered. Ethical approval for the study was granted by the institutional ethics and welfare committee (URN SR2021‐0148).

### 2.2 Criteria for case inclusion

Data from the questionnaire was compiled into a database and cases individually examined. Cats were considered to be eligible for inclusion if they presented with a leukopenia (<5.5 × 10^9^/L; or neutrophil concentration <2.5 × 10^9^/L) or thrombocytopenia (<150 × 10^9^/L), or a combination of these, with or without an anemia (hematocrit or packed cell volume <27%). Cats that presented with anemia without concurrent thrombocytopenia were excluded. If data on complete blood cell (CBC) analysis was not available, the response was excluded. Responses were included even if other sections were incomplete. Responses were excluded in the final analysis if they were received from countries other than the UK. Registered cats were excluded if follow‐up information provided by their primary care practice revealed an underlying disease process that could explain changes in blood cell concentrations (eg, neoplasia).

### 2.3 Feed sample analysis

Packages of pelleted dry feed of specific batch numbers of feeds that were frequently mentioned in the database and packages of commercial dry diets (“control samples”), which appeared infrequently in the database, were analyzed for trichothecene mycotoxin content including T‐2, HT‐2, and diacetoxyscirpenol (DAS) at the UK National Reference Laboratory for Mycotoxins and Plant Toxins in Food and Feed (FERA Science Ltd, York Biotech Campus, Sand Hutton, York, YO41 1LZ, UK). Trichothecene quantification was performed using liquid chromatography mass spectrometry.

### 2.4 Liver tissue analysis

Nontargeted chemical testing of postmortem liver tissue samples from affected and control cats (which were undergoing postmortem for a none‐related disease process) was performed using headspace gas chromatography‐mass spectrometry (HS‐GC‐MS), GC‐QTOF‐MS (gas chromatography/time of flight mass spectrometry) and LC‐TOF‐MS (liquid chromatography/time of flight mass spectrometry). Analysis was performed at a commercial laboratory (FERA Science Ltd, York Biotech Campus, Sand Hutton, York, YO41 1LZ, UK).

### 2.5 Statistical analysis

Following data checking for internal validity, identifying and correcting duplicate, incorrectly formatted and irrelevant data in Excel (Microsoft Office Excel 2013, Microsoft Corp), analyses were conducted using SPSS version 28.0 (IBM Corp). All continuous variables were nonnormally distributed and so were summarized using median and range. When data was compared between different groups Mann‐Whitney *U* test, chi‐square test and Fisher's exact test were used as appropriate.

Owner partial postcodes were geocoded using a free web‐based resource.^10^ A point map of cases was created in R statistical software (R version 4.2.0) using the “ggplot2” package.^11^

## 3 RESULTS

### 3.1 Demography

Between 24 May and 11 December 2021, 733 cats were logged via the questionnaire, 153 of those were excluded because of 1 or more of the following reasons: logged twice (n = 13), no CBC data available or not meeting inclusion criteria (n = 119); follow‐up data revealing underlying disease (n = 4); from outside the UK (n = 7); recent exposure to myelotoxic drug (n = 3); presented in 2020 (n = 7); and no entry of data (n = 2).

**FIGURE 1:**
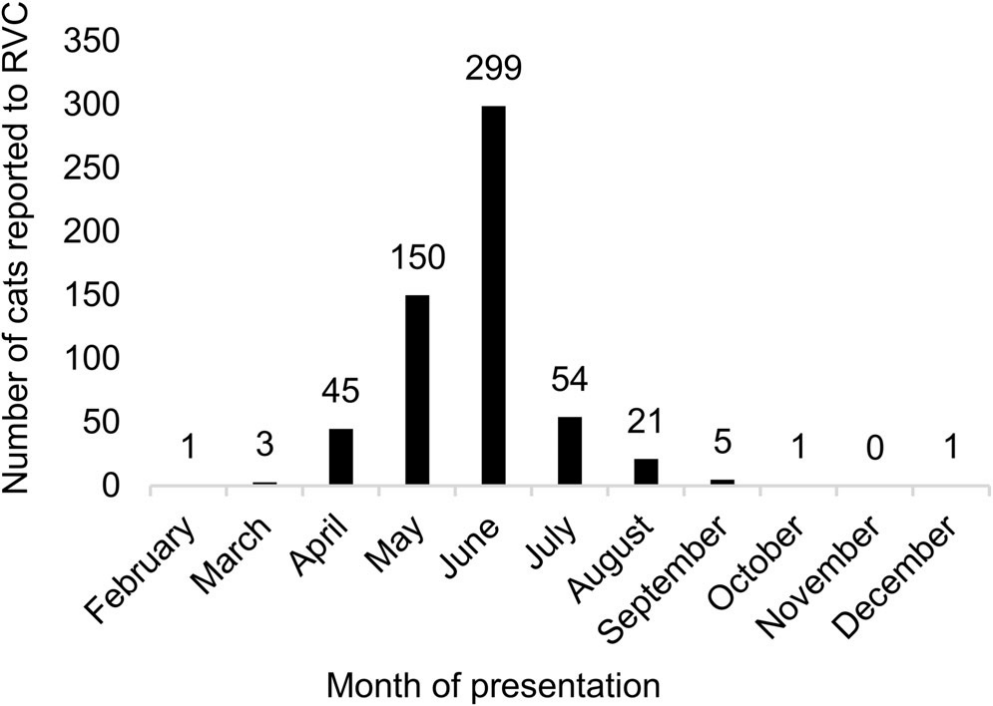
The number of cats presenting to veterinary practices with pancytopenia each month during the study period (n = 580)

The investigation included 580 cats presenting to 378 veterinary practices in the UK. The cats presented to veterinary practices between 26 February 2021 and 11 December 2021 (Figure 1). The median age at first presentation was 3.0 years (range, 0.0‐17.1). The most common breeds were domestic shorthair (364; 62.8%), ragdoll (39; 6.7%), British shorthair (36; 6.2%), domestic longhair (36; 6.2%) and crossbreeds (28; 4.8%). Of the cats, 217 (37.4%) were neutered males, 244 (42.1%) neutered females, 50 (8.6%) entire males, and 69 (11.9%) entire females.

### 3.2 Geographical distribution of affected cats

**FIGURE 2:**
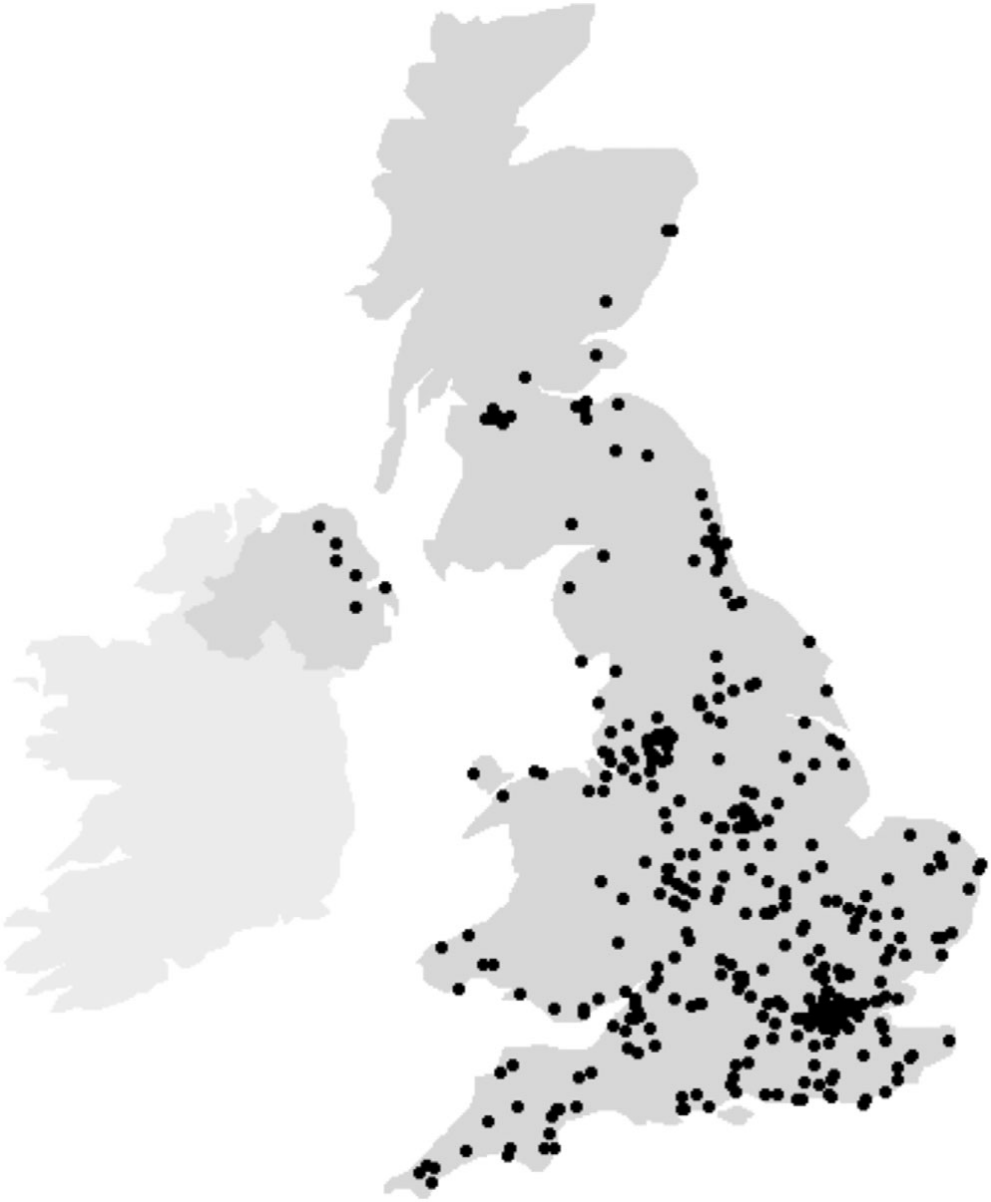
United Kingdom—Point map of cases using owner partial postcodes

Seven partial logged postcodes were incorrect and therefore excluded from case mapping. Owners of affected cats lived throughout the UK, although the majority were based in the Midlands, South‐East England, and London (Figure 2).

### 3.3 Cat characteristics, clinical signs, and diagnostics

There were 238 (41.0%) cats that were indoor only, while 342 (59.0%) had indoor and outdoor access. There were 239 (41.2%) cats from a single cat household, while 341 (58.8%) were from a multicat household. Of the cats from a multicat household, 167 (49.0%) cats were the only cat diagnosed with pancytopenia, while other cats in the household were diagnosed with pancytopenia in 174 (51.0%) cases. There were 258 (44.5%) cats that received flea or worming treatment within 2 months before presentation, while 262 (45.2%) received no treatment and this information was unknown in 60 (10.3%) cats. Of the cats receiving flea or worm treatment the most commonly used treatment brand was used in 21.7% (56 cats). There were 46 (7.9%) cats that received a new medication in the month before onset of clinical signs, while 524 (90.3%) received no new medication and this information was unknown in the remaining 10 (1.7%) cats. Vaccination within the month before pancytopenia presentation had been carried out in 53 (9.1%) cats, while 525 (90.5%) cats had not been vaccinated within the month before and vaccination status was unknown for the remaining 2 (0.3%) cats.

The median duration of clinical signs was 2.0 days (range, 0.0‐61.00). The most common clinical signs reported were lethargy (386; 66.6%), inappetence (304; 52.4%), pyrexia (220; 37.9%), petechiae (75; 12.9%), oral hemorrhage (64; 11.0%) and vomiting/gagging (62; 10.7%). The median total white blood cell concentration was 1.20 × 10^9^/L (range, 0.00‐24.00), median neutrophil concentration 0.47 × 10^9^/L (range, 0.00‐2.15), median platelet concentration (confirmed by slide examination) 8.00 × 10^9^/L (range, 0.00‐340.00) and median PCV 18.00% (range, 0.00‐59.40). The result of FIV testing was positive in 3 (0.52%) cats, negative in 284 (49.0%) cats and not performed in 293 (50.5%) cats. The result of FeLV testing was positive in 2 (0.3%) cats, negative in 284 (49.0%) cats and not performed in 294 (50.7%) cats. The result of FPLV testing was positive in 4 (0.69%) cats, negative in 63 (10.9%) cats and not performed in 513 (88.5%) cats. Of the 4 positive FPLV tests, 2 were weakly positive by polymerase chain reaction performed on a bone marrow aspirate sample only. Bone marrow examination revealed hypoplasia in 36 (6.2%) cats, aplasia in 18 (3.1%) cats, hyperplasia in 2 (0.3%) cats and was not performed in 524 (90.3%) cats. Overall, 367 (63.3%) affected cats died, while 210 (36.2%) cats were alive at time of follow‐up, with this information not reported in 3 (0.52%) cats. COVID‐19 status of the family was unknown for 241 cats (41.6%), negative for 330 cats (57.0%) and positive for 9 cats (1.6%).

### 3.4 Diet

**FIGURE 3:**
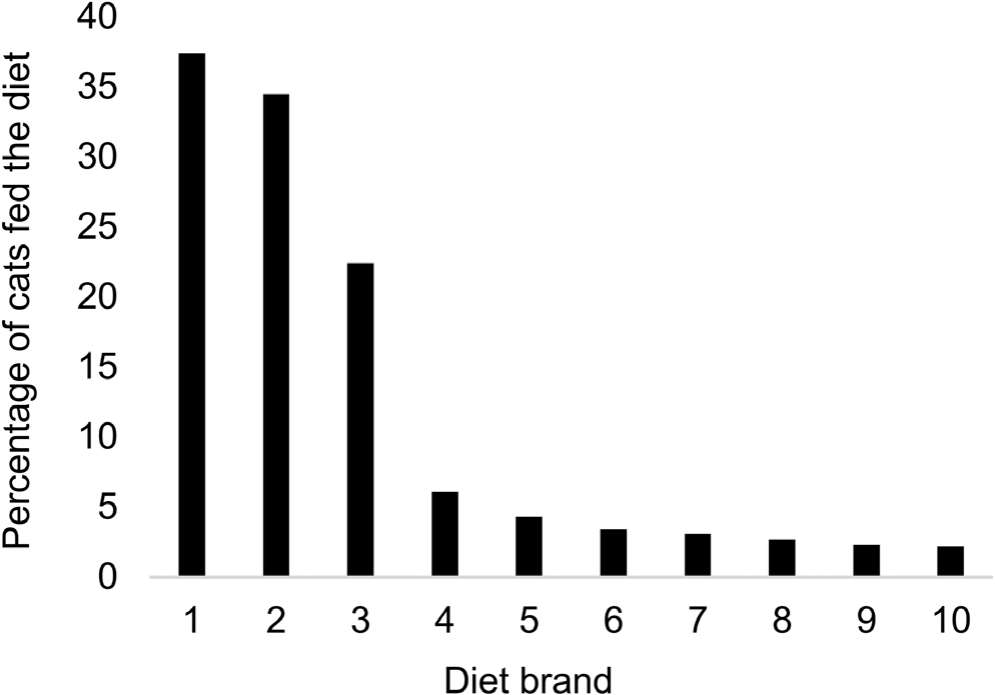
Most common diet brands (%) fed to affected cats with brand information recorded (n = 554)

In total, 44 diet brands were fed to the 554 cats with brand information recorded, with 3 diet brands (brands A, B, and C) predominating (Figure 3). These 3 brands were recalled from the market on 16 June 2021.^12^ There were 500 (86.2%) cats fed a recalled product, while 54 (9.3%) cats were not fed a recalled product, with diet information unknown in 26 (4.5%) cats. Of the cats that were not fed a recalled product, 38 (70.4%) had indoor and outdoor access, while 16 (29.6%) were indoor only. There had been a recent diet change in 106 (18.3%) cats, no recent change in 472 (81.4%) cats, while this information was not recorded in 2 (.34%) cats. Of the cats that were not fed a recalled product, a recent diet change was reported for 11, including 4 of the 16 indoor cats.

### 3.5 Comparison of cases presenting on or before food recall or after food recall

**FIGURE 4:**
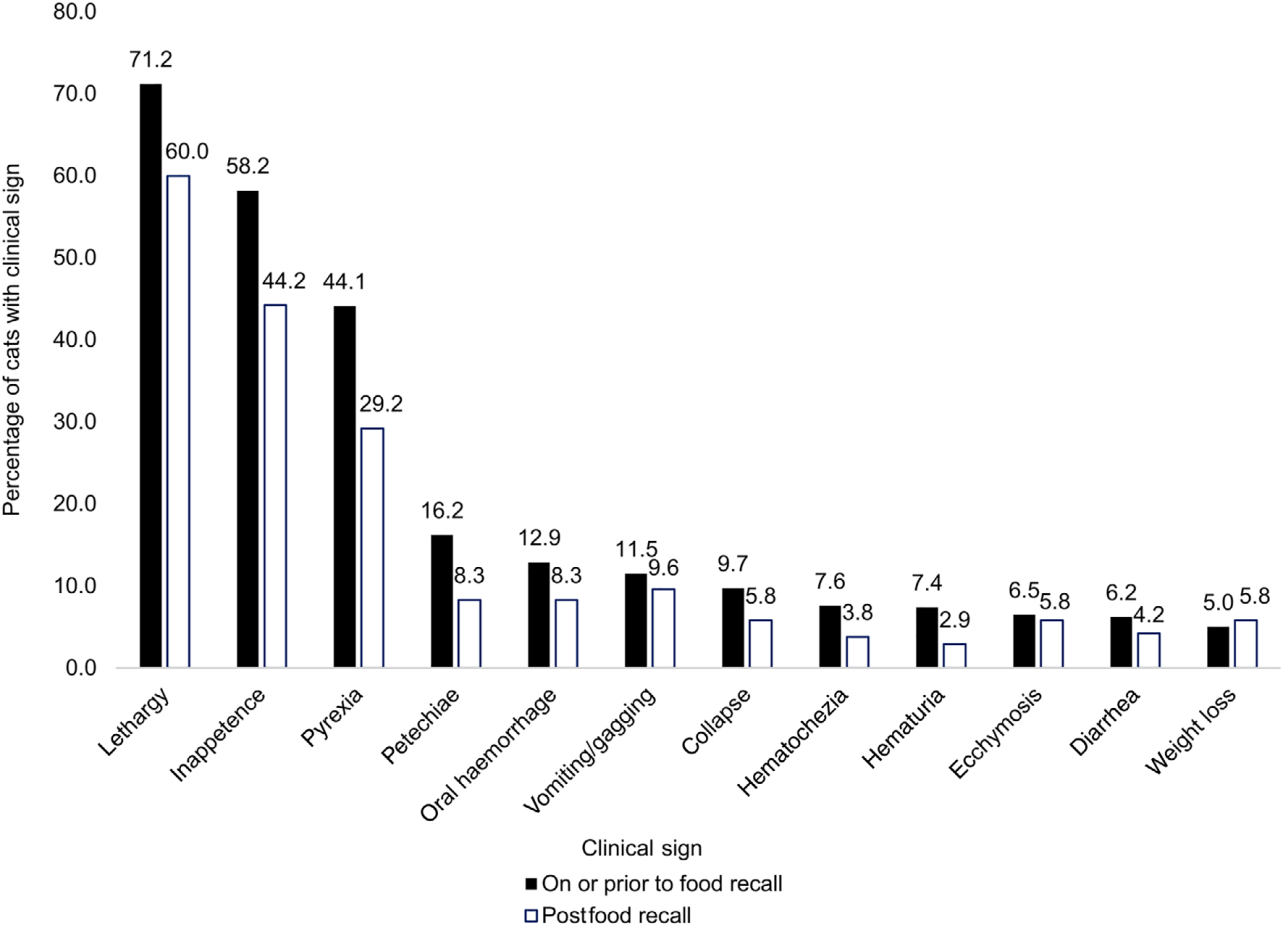
Most common clinical signs (%) in cats presenting on or before the date of food recall (n = 340) and after food recall (n = 240)

There were 340 (58.6%) cats that presented on or before the date of food recall (16 June 2021), while 240 (41.4%) cats presented after the date of food recall. The 10 most common clinical signs in cats presenting on or before the date of food recall and the 10 most common clinical signs in cats presenting after food recall were combined, resulting in an overall list of 12 clinical signs. Cats presenting on or before food recall had a higher proportion of 11/12 (91.7%) clinical signs, while cats presenting after food recall had a higher proportion of 1/12 (8.3%) clinical signs (Figure 4). On or before food recall, 2 cats (0.6%) were presented by the owner as they were fed a recalled diet, while after food recall 37 cats (15.4%) were presented by the owner for the same reason. In addition, 8 cats (2.4%) presenting on or before food recall were presented as a housemate was diagnosed with pancytopenia, while 15 cats (6.3%) presenting after food recall were presented as a housemate was diagnosed with pancytopenia.

The duration of clinical signs, total white blood cell concentration, neutrophil concentration, platelet concentration, PCV and case fatality were compared between cats presenting on or before the date of food recall and after food recall (Table 1).

**TABLE 1:** Comparison of duration of clinical signs, hematological abnormalities and mortality in cats presenting on or prior to the date of food recall (n = 340) or postfood recall (n = 240)

Value under comparison	On or prior to food recall	Postfood recall	*P*‐value
Median duration of clinical signs (days)	2.00 (range, 0‐31) (n = 338)	1.00 (range, 0‐61) (n = 240)	.03
Median total white blood cell concentration (×10^9^/L)	0.90 (range, 0.00‐24.00) (n = 325)	1.80 (range, 0.00‐17.90) (n = 236)	<.001
Median neutrophil concentration (×10^9^/L)	0.08 (0.00‐2.15) (n = 6)	0.70 (0.04‐1.95) (n = 12)	.11
Median platelet concentration (confirmed by slide examination) (×10^9^/L)	6.00 (0‐340) (n = 214)	15.00 (range, 0‐260) (n = 147)	<.001
Median PCV (%)	15.55 (range, 0.00‐55.00) (n = 327)	22.0 (range, 5.40‐59.40) (n = 238)	<.001
Mortality (%)	76.2 (n = 259)	45.0 (n = 108)	<.001

Of cats presenting on or before the date of food recall with diet information available, 298/319 (93.4%) were fed a recalled diet, while 21 (6.6%) were not fed a recalled diet. This differed to cats presenting after food recall, whereby a lesser proportion (202/235; 86.0%) of cats were fed a recalled diet, while a greater proportion (33; 14.0%) were not fed a recalled diet.

### 3.6 Feed analysis

The trichothecene feed analysis revealed T‐2/HT‐2 sum concentration higher than the recommended guidance value in 5 of 7 analyzed dry feed packages of recalled brands (Table 2).^13^ Two of 3 control samples did not contain any detectable concentration of T‐2/HT‐2 and 1 of the control samples contained detectable T‐2/HT‐2 but at a concentration below the recommended guidance values (Table 2).^13^ DAS was also detectable in all 7 samples of recalled brands but not in any of the control samples (Table 2).

**TABLE 2:** Concentration of trichothecene mycotoxins detected in feeds

	Trichothecenes (μg/kg)			
Feed	T‐2 toxin	HT‐2 toxin	T‐2/HT‐2 toxin sum	Diacetoxyscirpenol
Sample A	18	64.5	82.5	194
Sample B	<5	6.1	6.1	62.6
Sample C	9.2	41.1	50.3	75.6
Sample D	39.3	178	217.3	364
Sample E	27.9	127	134.9	261
Sample F	<5	13	13	57.1
Sample G	33.2	143	176.2	305
Control A	<5	<5		<5
Control B	<5	<5		<5
Control C	7.3	9.6	16.9	<5

### 3.7 Liver tissue analysis

The nontargeted chemical testing of postmortem liver samples found no single feature present in all livers from 5 affected cats. There was no evidence of any known toxin or metabolite of any known toxin. Specific targeted metabolite searches found peaks indicative of mycotoxin exposure, specifically T‐2 toxin, and DAS.

## 4 DISCUSSION

The clinical pathological findings of the cats affected in this outbreak were most consistent with an aplastic pancytopenia (formerly also called aplastic anemia), which is characterized by cytopenias of erythrocytes, leukocytes and platelets in the peripheral blood combined with a hypo‐ or acellular bone marrow with the marrow space being replaced by adipose tissue.^14^ Aplastic pancytopenia is a rare disorder in cats which can be caused by destruction of bone marrow stem cells, genetic mutations causing inadequate stem cell function or hemopoietic microenvironment disorders.^14^ Idiopathic aplastic pancytopenia is described in people but this has not been reported in cats.^14^ Reported toxic causes of aplastic pancytopenia in cats include administration of phenobarbitone, griseofulvin, trimethoprim/sulphonamide and various chemotherapeutic agents.^14^, ^15^ Infectious causes include feline leukemia virus, feline immunodeficiency virus, *Ehrlichia* spp. and parvovirus.^14^ Cobalamin deficiency can cause aplastic pancytopenia in cats.^5^

There was no recognized infectious cause or obvious link to medication or vaccination detected in the data gathered, but 3 reported brands of cat food; which were all made at the same factory; were noted as being fed to the majority (86.2%) of the cats. This led to a voluntary removal of brand A from retailers on 11 June 2021 and a full product recall of brands A, B, and C on 16 June 2021.^12^ Industry figures detailing sales of various brands are difficult to obtain, but brands A, B, and C are not recognized as common diets for cats in UK and of the 3 brands, only brand A is listed in the top 10 dry cat food average monthly sales for Amazon, placed in 7th position with sales of less than a quarter of the most popular brand.^16^, ^17^ Fifty‐four cats (9.3%) were recorded to have been eating a nonrecalled brand. It is possible that some of these cats might never have been exposed to the suspected diets as a minority were reported as indoor cats only. It is also possible that some registered cats had an underlying disease that was not identified because of lack of investigations, that some of the cats accessed a recalled brand outside the home and finally that some cats could have had a recent diet change with the veterinarian only noting the brand that was being fed at the time of presentation without further verification whether a diet change had occurred or not. The latter theory is also suspected to be the reason for the significantly lower proportion of cats being fed a recalled diet after the recall had occurred.

The strong epidemiological link to feed—with over 86% of affected cats being exposed to at least 1 of the recalled diets—suggested either a feed deficiency or intoxication. Diagnostic tests performed on individual cats showed no evidence of vitamin deficiency or heavy metal intoxication.^18^ The group A tricothecene mycotoxins T‐2 and HT‐2 (a metabolite of T‐2), produced by *Fusarium* spp. fungi, cause alimentary toxic aleukia in humans in which pancytopenia and bleeding because of bone marrow toxicosis are seen.^19^, ^20^, ^21^ T‐2 and HT‐2 trichothecene mycotoxins are fatal when administered enterally and parenterally to cats, with clinical signs before death including lethargy, anorexia, bloody diarrhea and weight loss consistent with the clinical signs seen in our cohort.^22^, ^23^, ^24^ There is low cellularity of bone marrow on postmortem examination of cats administered T‐2 toxin.^23^ Food aversion is commonly reported in several species when food is contaminated with T‐2 and HT‐2 and inappetence was a frequent clinical sign reported in the cats affected in this outbreak.^25^

Although strict controls are in place for maximum levels of trichothecene contamination of human foodstuffs there is no legal limit for pet foods.^26^ Because of lack of data on the specific biotransformation and toxicodynamics in cats, a “no observed adverse effect level” (NOAEL) or a “lowest observed adverse effect level” (LOAEL) for trichothecenes in cats has not yet been established.^27^ European Union guidance for a maximum combined T‐2 and HT‐2 level of 50 μg/kg in dry cat food is based on experimental studies.^13^, ^22^, ^23^, ^24^ This level is lower than that for other animal feeds as it is widely recognized that cats are extremely susceptible to T‐2 toxin induced hematotoxicity compared to other species which is postulated to be because of their decreased ability to form glucuronide conjugates.^27^, ^28^ The combined levels of T‐2 and HT‐2 were greater than 50 μg/kg in 5 of the 7 samples of recalled foods, but none of the control diets. This is consistent with a study of 60 cat foods where the range of combined T2 and HT‐2 concentrations was 1.24 to 7.98 μg/kg.^25^ This finding and the marked similarity between the clinical signs of this cohort of cats and those reported in experimental studies of cats suggests that trichothecene contamination of cat food brands A, B and C is a possible cause of this pancytopenia outbreak.^22^, ^23^, ^24^

Trichothecene diacetoxyscirpenol (DAS) was detected in all food samples of recalled foods but none of the control brands. DAS is a group A trichothecene (as are T‐2 and HT‐2) with a similar structure to T‐2 and its main toxic effects include emesis and hematotoxicity.^29^, ^30^ As for T‐2/HT‐2 glucuronide conjugation is 1 of the main metabolization pathway for DAS, lacking in cats.^30^ In mice, the LOAEL of DAS administered orally to cause anorexia is 5 times greater than for either T‐2 or HT‐2.^31^ In dogs, NOAELs for DAS are based on laboratory studies for both chronic and acute exposure but are not reported in cats.^32^, ^33^ There is no data describing the levels of DAS found in cat food, but estimated exposure levels based on concentrations in cereal grains and their relative proportions in diets suggest 95th percentile diet concentration of 13 μg/kg in dry cat food and this is therefore thought to be a safe level.^30^ The levels of DAS detected in the recalled foods tested were far higher than this 95th percentile and it is suspected that it could have caused adverse effects.

T‐2 and HT‐2 are considered to be cumulative when assessing risk of toxicosis in feed.^17^ Also, dietary co‐exposure to multiple toxins produced by *Fusarium* spp. is thought to be common,^30^ with the effects of T‐2 and DAS being additive for oral lesions and synergistic in decreasing egg production in laying hens.^34^ Therefore, the combination of contamination of the tested cat foods with all 3 mycotoxins could have led to a greater adverse effect than that would be predicted by considering the concentration of each mycotoxin individually.

The levels of T‐2/HT‐2 and DAS, although generally increased compared to the control samples and those reported in the literature, were variable between the samples of recalled brands. Ideally, a large number of small samples are taken to gather an impression of an average concentration in a product.^35^ This variation in concentration could, at least in part, account for the variation in severity of clinical signs seen in affected cats and could have meant that some cats eating the recalled brands were unaffected.

As with previously described outbreaks of disease, the initial suspicion occurred because of practitioners noting an increase in cats presenting with similar clinical signs.^4^, ^36^, ^37^ The analysis of case numbers of pancytopenia of unknown cause in cats presenting to the authors' hospital in the previous 5 years supported the tentative postulate that an outbreak was occurring. Small animal epidemiological tools do exist to monitor disease trends in the cat population presenting to UK veterinary practices.^38^, ^39^ However, even in comparatively large disease outbreaks such as this 1, the number of cases could be too low to determine an increase in case numbers with these tools, particularly if presenting signs are vague and specific keywords or treatments are not always present in the clinical record. Even if the extent of an outbreak can be investigated in this way, suspicion needs to be raised initially to allow further investigation.^40^

There is currently no clear pathway to deal with a suspected nonnotifiable disease outbreak in cats or dogs in the UK. This can lead to a slower than desirable response to an outbreak such as this 1. The financial restrictions many clients might face can prevent full investigation into the cause of disease, meaning whether an outbreak has an infectious or toxic (or other) cause could be difficult to determine. If the cause of the outbreak is unknown, leadership and management of investigation is even less evident. If a further cat food mycotoxin contamination event were to occur, it is hoped that the information provided here would allow a more rapid recognition of the possible cause and action by the relevant authorities.

Despite a major effort and support received for publicizing the investigation to call for active participation of veterinary surgeons in first opinion and referral practice UK wide, we suspect that many veterinary surgeons remained unaware of the investigation, resulting in an unknown number of affected cats not being included in our database. Furthermore, cats presenting with consistent clinical signs and history, were not included if a complete blood count was not performed.

Once a possible association with diets was made, this allowed notification of the Food Standards Agency to lead the investigation which led to a voluntary full product recall of brands A, B and C. Various disease outbreaks secondary to commercial pet food ingestion are reported around the world, including acute kidney injury because of melamine and cyanuric acid feed contamination,^41^, ^42^ acute kidney injury and hypercalcemia because of excessive vitamin D feed supplementation,^43^ dietary associated acquired renal proximal tubulopathy,^44^ hepatotoxicosis because of indscopine feed contamination,^45^ dietary associated acquired megaesophagus^46^ and demyelination secondary to irradiation of feed.^47^ It should be remembered these outbreaks are rare and the pet food industry is held to high standards in the UK. As home‐produced diets can also cause disease in pets,^48^ veterinary surgeons should take care to reassure their clients that commercial food is generally safe.^46^ However, these recurrent world‐wide events also highlight the urgent need for establishing a better framework for investigating suspected foodborne disease outbreaks, with a possible solution modeled in Australia with the Pet Food Adverse Event System of Tracking (PetFAST). This is a veterinary reporting system to track suspected adverse events related to pet food, pet meat and treats which is used to identify possible patterns indicating a problem. It is a joint initiative of the Australian Veterinary Association (AVA; https://www.ava.com.au/) and the Pet Food Industry Association of Australia (PFIAA; http://www.pfiaa.com.au/) and could be replicated in other countries.

There were a number of limitations in this investigation. The data obtained was from a wide variety of veterinary surgeons with varying levels of investigations performed for the individual cat but also with varying levels of quality of the provided data (eg, details on diets). Ideally, a control group would have been concurrently created, to have presentation location, time and signalment matched controls for each case. However, because of the fast‐paced and severe nature of the outbreak, this did not occur and there was concern that this would have significantly increased the work of veterinarians submitting cases, likely leading to decreased registration. Ideally these control cats should have also had a complete blood count performed, though likely this would have led to difficulty in recruiting suitable controls.

Although there is a strong association between the ingestion of previous batches of brands A, B, and C and the occurrence of pancytopenia, diet cannot be definitively stated to be the cause as there is no LOAEL established for T‐2, HT‐2, and DAS in cats. No specific source of mycotoxin was established, although it is recognized that many possible feed ingredients could be responsible in cat food manufacture, particularly wheat, oat and maize; but also root vegetables.^49^ Given the high proportion of affected cats known to be consuming the 3 brands, their common manufacturing source, the detection of T‐2 and HT‐2 in the feeds at levels much higher than those previously reported in cat feeds (and above that recommended by the European Union), the consistency between the clinical signs seen with T‐2/HT‐2 intoxication in various species including cats and those seen in our cohort, the decreased severity of clinical signs (likely because of decreased toxin exposure) and eventual resolution of the outbreak when the 3 brands were withdrawn from the market, it is reasonable to propose that trichothecene contamination of the recalled food brands was the cause of this pancytopenia outbreak in cats.

In conclusion, trichothecene intoxication should be considered as a possible differential diagnosis for a cat presenting with pancytopenia of unknown cause and if an outbreak were to occur in the future, rapid investigation of feed should be undertaken. This investigation has highlighted the need for introducing standardized testing of cat foods for mycotoxin contamination across the pet food industry and following this the establishment of NOAELs for T2/HT2 and DAS in cats through greater monitoring of pet feed levels.

## CONFLICT OF INTEREST DECLARATION

Authors declare no conflict of interest.

## OFF‐LABEL ANTIMICROBIAL DECLARATION

Authors declare no off‐label use of antimicrobials.

## INSTITUTIONAL ANIMAL CARE AND USE COMMITTEE (IACUC) OR OTHER APPROVAL DECLARATION

Approved by the Royal Veterinary College institutional ethics and welfare committee (URN SR2021‐0148).

## HUMAN ETHICS APPROVAL DECLARATION

Authors declare human ethics approval was not needed for this study.

## Supporting information


**Data S1**. Supporting Information
