# Supplementary material for: An investigation into an outbreak of pancytopenia in cats in the United Kingdom
Source: J Vet Intern Med. 2023 Jan 7;37(1):117–25. doi: 10.1111/jvim.16615 (PMC9889669; doi:10.1111/jvim.16615)
Supplement: Supplementary file 1 — Data S1. Supporting Information [file JVIM-37-117-s001.pdf]

Page 1: Feline Pancytopenia

Thank you for taking the time to complete this questionnaire.

The current marked and unusual increase in feline pancytopenia cases is very concerning.

This questionnaire consists of several questions about clinical presentation and clinicopathological data to ensure correct case selection, followed by questions about different aspects of the patient's demographic characteristics, vaccine and drug history, lifestyle, diets, and surroundings.

The information provided will be used to try to identify a common factor among cases so that we can identify any underlying cause.

Please complete this survey separately for each affected cat.

The information provided may be used for research purposes, but we will not use or store personal information that might identify the client. If you have any questions, please do not hesitate to contact us.

*1.*Please confirm that you are a veterinary surgeon or veterinary nurse **If you are the owner of an affected cat, please ask your veterinary surgeon to complete this survey** *Required*

- ○ Yes
- ○ No

Page 2: Presentation

*2.*What is the name of your practice? *Required*

[ ]

*3.*What is the post code of your practice? *Required*

[ ]

*4.*Without telling us the name of the client, please tell us a unique identifier for the case (E.g. a code from your records management system) *Required*

[ ]

Page 3: Clinical presentation

5. When did the owner first notice clinical signs? *Required*

Dates need to be in the format 'DD/MM/YYYY', for example 27/03/1980.

(dd/mm/yyyy)

6. When was the cat first presented to your practice for investigations? *Required*

Dates need to be in the format 'DD/MM/YYYY', for example 27/03/1980.

(dd/mm/yyyy)

7. What were the clinical signs and physical examination abnormalities? *Required*

8. What breed is the cat? *Required*

9. What is the sex and neuter status of the cat? *Required*

- Female entire
- Female neutered
- Male entire
- Male neutered

10. What is the date of birth of the cat? *Required*

Dates need to be in the format 'DD/MM/YYYY', for example 27/03/1980.

(dd/mm/yyyy)

11. What is the first part of the post code of the owner? (E.g. AL9) *Required*

Page 4: Clinical pathology

12. Which haematological abnormalities does the cat have? *Required*

- [ ] Anaemia
- [ ] Thrombocytopenia
- [ ] Neutropenia
- [ ] Low total white blood cell count

13. What is the total white blood cell count? *Required*

14. What is the platelet count? *Required*

15. How was the platelet count obtained? *Required*

- ( ) Machine count only
- ( ) Manual count from slide only
- ( ) Machine count confirmed by slide examination

16. What is the PCV? *Required*

17. What is the result of FIV testing? *Required*

- ( ) Positive
- ( ) Negative
- ( ) Not performed

18. What is the result of FeLV testing? *Required*

- ( ) Positive
- ( ) Negative
- ( ) Not performed

*19.*What is the result of testing for feline panleukopenia/feline parvovirus? *Required*

- Positive
- Negative
- Not performed

*20.*What is the result of bone marrow examination? *Required*

- Aplasia
- Hypoplasia
- Hyperplasia
- Other
- Not performed

Page 5: Patient characteristics

*21.*Where does the cat spend time? *Required*

- Indoor only
- Indoor and outdoor

*22.*Are there any other cats in the household? *Required*

- Yes
- No

*23.*If yes, please indicate how many other cats are in the household and if any is a new cat in the house

*24.*Are any other cats in the household suffering from pancytopenia? *Required*

- Yes
- No

25. Are there any other pets in the household apart from cats? Please list all below. *Required*

26. Does the cat with pancytopenia take any long-term medications? Please list below. *Required*

27. Has the cat with pancytopenia received any flea or worming treatment in the past 2 months? Please list all below, with batch numbers if known. *Required*

28. Has the cat with pancytopenia received any new medications in the month before the onset of clinical signs? Please list all below. *Required*

29. Has the cat with pancytopenia been vaccinated in the past month? *Required*

- Yes
- No

30. What is the cat's current diet? Please list all products with batch numbers if possible and including treats. *Required*

31. Has there been any recent change in diet, including treats? *Required*

- Yes
- No

32. Does the cat receive any dietary supplements? Please list all below. *Required*

33. Has any person in the household tested positive for covid-19 in the past 2 months? *Required*

- Yes
- No
- Don't know

Owner prefers not to say

34. Is the cat still alive? *Required*

Yes

No

35. If the cat has died or been euthanised, when did this happen?

Dates need to be in the format 'DD/MM/YYYY', for example 27/03/1980.

(dd/mm/yyyy)

36. What brand of cat litter does the cat use? *Required*

Page 6: Further contact

37. Would you be happy for us to contact you for further information or details of the follow-up of the case? *Required*

Yes

No

38. If yes, please give your name or the name of a suitable contact at your practice

39. If yes, please give your email address or phone number

40. Would the owner be happy for us to contact them directly? *Required*

Yes

No

41. If yes, please give the owner's contact details (email address preferred).

Page 7: Final page

Thank you for participating in this survey. If you know of any colleagues who have seen a cat with pancytopenia recently, please encourage them to complete this survey too.
